# The effect of oxygen concentration on the speciation of laser ablated uranium

**DOI:** 10.1038/s41598-022-07834-9

**Published:** 2022-03-07

**Authors:** Mark A. Burton, Alex W. Auner, Jonathan C. Crowhurst, Peter S. Boone, Lauren A. Finney, David G. Weisz, Batikan Koroglu, Igor Jovanovic, Harry B. Radousky, Kim B. Knight

**Affiliations:** 1 Lawrence Livermore National Laboratory, 7000 East Ave, Livermore, CA 94550 USA; 2 Department of Nuclear Engineering and Radiological Sciences, University of Michigan, 2355 Bonisteel Blvd, Ann Arbor, MI 48109 USA

**Keywords:** Analytical chemistry, Physical chemistry

## Abstract

In order to model the fate and transport of particles following a nuclear explosion, there must first be an understanding of individual physical and chemical processes that affect particle formation. One interaction pertinent to fireball chemistry and resultant debris formation is that between uranium and oxygen. In this study, we use laser ablation of uranium metal in different concentrations of oxygen gas, either ^16^O_2_ or ^18^O_2_, to determine the influence of oxygen on rapidly cooling uranium. Analysis of recovered particulates using infrared absorption and Raman spectroscopies indicate that the micrometer-sized particulates are predominantly amorphous UO_x_ (am-UO_x_, where 3 ≤ x ≤ 4) and UO_2_ after ablation in 1 atm of pure O_2_ and a 1% O_2_/Ar mixture, respectively. Energy dispersive X-ray spectroscopy (EDS) of particulates formed in pure O_2_ suggest an O/U ratio of ~ 3.7, consistent with the vibrational spectroscopy analysis. Both am-UO_x_ and UO_2_ particulates convert to α-U_3_O_8_ when heated. Lastly, experiments performed in ^18^O_2_ environments show the formation of ^18^O-substituted uranium oxides; vibrational frequencies for am-U^18^O_x_ are reported for the first time. When compared to literature, this work shows that cooling timescales can affect the structural composition of uranium oxides (i.e., crystalline vs. amorphous). This indicator can be used in current models of nuclear explosions to improve our predicative capabilities of chemical speciation.

## Introduction

Following a nuclear event, fallout particulates are formed from and affected by gas-phase chemical reactions and micro-physical processes involving hot nuclear material and the environment^1,2^. Understanding reactions between device materials, such as uranium, and the surrounding environment under these conditions is essential to improve nuclear debris formation models developed in the early days of nuclear testing^3–5^. Models based on empirical data from historic nuclear tests aim to predict the chemical speciation and evolution of a nuclear explosion^6,7^ but reflect testing performed under ideal conditions across a limited set of environments. To more broadly predict the behavior of actinides and their incorporation into debris from diverse potential nuclear events, new experimental inputs are needed to determine key system sensitivities such as local environment conditions. Here, we utilize laboratory scale experiments to provide new insights on the interaction of oxygen and uranium relevant to understanding debris formation and chemical speciation dynamics within an evolving nuclear fireball.

The interaction of uranium and oxygen is one system pertinent to fireball chemistry and resultant debris formation^1,8–10^. The uranium–oxygen system is complicated to study as there are numerous stable stoichiometric phases (with O/U ranging from 2 to 4) with multiple polymorphs for a given stoichiometry (e.g., amorphous vs crystalline UO_3_), the possibility of sub- and super stoichiometric phases, and the ability for phases to interconvert depending on the conditions (e.g., temperature, hydration, and gaseous environment)^11–14^. For example, thermally dehydrating UO_4_∙4H_2_O (“studtite”) will form amorphous UO_3-4_ which can then be heated at ~ 550 °C to form crystalline α-UO_3_^15^ or at ~ 650 °C to form α-U_3_O_8_^16^. Additionally, α-U_3_O_8_ can in turn either be heated in the presence of NO_2_ (250–375 °C) to form ε-UO_3_ or 40 atm O_2_ (500–550 °C) to form β-UO_3_^15,17^. In order to relate to nuclear fireball chemistry, however, reactions between uranium and oxygen must be studied while hot (several thousand Kelvin) vapor-phase material cools, instead of how materials are heated.

One approach to emulate the temperature conditions relevant to study nuclear fireball chemistry in the laboratory is with plasma discharges or high-power lasers^18–23^. Such studies, for instance, have shown the formation of gas-phase UO after laser ablation (LA) of uranium^24–29^. Furthermore, several uranium oxides have been isolated in cold matrices (e.g., N_2_, Ar, and Kr) after LA of uranium or thermal vaporization of uranium or UO_2_^30–32^. These studies show the formation of UO, UO_2_, UO_3_, (UO_2_^+^)(O_2_^–^), (UO_2_^2+^)(O_2_^2–^), and UO_3_–O_2_. Although the cooling timescales between LA and nuclear fireball events are different (microseconds verses seconds varying as a function of yield, respectively), the plasma temperature produced by LA and ability for ablated material to vaporize^26–28,33^ is similar to nuclear fireball conditions. Thus, LA could have relevance to understanding the chemical processes occurring on short timescales within a nuclear event.

In addition to temperature, it is also important to understand how uranium reacts in atmospheres with both abundant and scarce amounts of oxygen as interactions with the environment (e.g., from shockwaves) are likely to affect local oxygen availability and distribution for a nuclear fireball. Recent studies by Koroglu et al.^1,23^ have used an inductively coupled plasma (ICP) to investigate the evolution of uranium oxidation as a function of temperature from 5000 to 1000 K. These authors found gas-phase UO forms shortly after the torch but was no longer detected below 2000 K, which was attributed to the formation of higher-oxides. Particulates recovered at 1000 K were determined to be fcc-UO_2_ or α-UO_3_, depending on the input oxygen concentration, demonstrating that oxygen availability will influence the speciation, and thus chemical volatility, of uranium in such environments.

These studies, however, used a uranium nitrate solution as the metal precursor meaning experiments performed under relatively low oxygen conditions still contained a large amount of oxygen, as well as other species such as nitrogen and hydrogen. LA of uranium metal in specific oxygen concentrations can be used as a complementary technique to achieve a more controlled atmosphere. As this approach can be performed inside a sealed vacuum chamber, isotopic reagents (e.g., ^18^O_2_) can be more readily used. Furthermore, although the ICP apparatus used by Koroglu et al.^1,23^ can study vapor phase cooling over milliseconds, this timescale is still orders of magnitude away from the cooling of a nuclear fireball. As it is difficult to achieve high temperature cooling over seconds, data collected at microseconds (LA) and milliseconds (ICP) could be used to extrapolate to longer cooling timescales. Although the measured physical quantities obtained from two different setups will be valid over certain cooling regimes, care must be taken when combining data sets between techniques with different starting temperatures and thermodynamics, introduction of species, and gaseous surroundings.

Here we present results from benchtop laser ablation experiments on uranium in environments with high or low concentrations of oxygen where particulates are collected and analyzed with various spectroscopies. We use the results of these experiments to show that the nature of formed particulates is sensitive to the available oxygen and varies from a highly oxidized amorphous form (am-UO_x_ where 3 ≤ x ≤ 4) to crystalline UO_2_. We find that particulate formation is additionally sensitive to heat and, in the case of am-UO_x_, humidity. This work, aimed at understanding high temperature reactions between uranium and controlled atmospheres, provides a crucial first step for understanding particle formation relevant to nuclear forensics. The results obtained here can also be used as the basis for studying the speciation of more complex systems, e.g., uranium compounds or alloys, which are often used in nuclear power plants as fuel.

## Experimental

**Figure 1:**
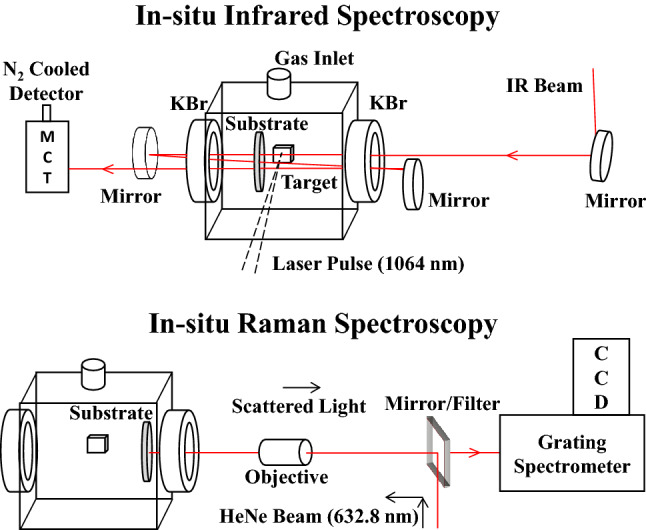
Schematic of the ablation chamber and configurations with in-situ infrared (top) and Raman (bottom) spectroscopies. Shown is a 2.75″ CF 6-way cube with two KBr window flanges and one gas inlet flange. The chamber also consists of one sapphire window flange (for the laser pulse), one translation arm flange (to hold the uranium target), and one blank flange (as a supporting base) not shown for clarity. The total length is ~ 6 inches along the IR beam axis. Particulates formed during the ablation process are collected on a KBr substrate positioned close to the ablation plume. Infrared light, guided by gold-coated focusing mirrors, makes multiple passes through the substrate to obtain an IR absorption spectrum. Tilting the chamber moves the KBr substrate towards the external window allowing for in-situ Raman spectra to be measured.

A diagram of the ablation chamber is shown in Fig. 1, which depicts the setup for in-situ IR spectroscopy (top) and Raman spectroscopy (bottom); “in-situ” and “ex-situ” refers to inside and outside the chamber, respectively. The chamber consists of two IR transparent viewports (with removable 25 mm diameter × 5 mm thick KBr windows) perpendicular to the IR radiation axis and a sapphire viewport parallel the axis (not shown) used to introduce the laser pulse. Vacuum fittings are connected to the top of the chamber to introduce and control the atmosphere. Particulates formed during LA are collected on a 25 mm diameter × 2 mm thick KBr substrate placed inside the chamber, supported by an aluminum holder. The depleted uranium target is adhered with carbon tape to a translation flange, opposite of the sapphire viewport, which is linearly adjusted to optimize particulate placement onto the collection substrate. This substrate was ~ 1 cm away from the ablation plume. Externally, gold coated mirrors are used to focus the IR beam through the chamber, making three passes through the substrate, before reaching a liquid N_2_ cooled broadband (12,000–420 cm^−1^) mercury-cadmium telluride (MCT) detector. A Bruker Invenio-R Fourier transform infrared (FTIR) spectrometer, equipped with a SiC globar source, was used to collect infrared spectra.

For each experiment, the uranium target was polished to 1 μm surface roughness, quickly transferred to the reaction chamber, and placed under vacuum. The polished surface interacted with air for only a few minutes and it is believed that any oxidation during this time would not affect the conclusions drawn from this experiment. After evacuation to < 100 mTorr, the chamber was filled with either 1 atm of pure O_2_ (99.98%, Sigma Aldrich), pure ^18^O_2_ (99%, Sigma Aldrich), or 1% O_2_/Ar (Matheson). Once the chamber was aligned and a background IR spectrum taken, the ablation proceeded using a 1064 nm Nd:YAG laser (Quantel ULTRA 100) with a pulse duration of ~ 8 ns (50 mJ) and operated at 20 Hz. A 10 cm focusing lens was used to reduce the beam diameter. The uranium was ablated continuously for 15 min while occasionally moving the laser beam via optics to prevent creating deep craters. An IR absorption spectrum was then taken consisting of 30 averaged scans at 1 cm^−1^ resolution from 400 to 8000 cm^−1^; each scan took less than one minute.

After in-situ IR spectra were obtained, the chamber was evacuated to prevent possible further reactions with O_2_ and positioned for Raman spectroscopy. The Raman measurements employed a HeNe laser (632.8 nm) to excite the Raman process. Mitutoyo apochromatic microscope objectives were used to focus the laser light onto the sample particulates as well as collect scattered light. The Raman system includes an embedded microscope which was used to locate the particulates. In-situ spectra were collected by tilting the chamber such that the aluminum holder would slide towards the external window bringing the particulates within range of the working distance of the chosen objective (e.g., 20 mm with 20x); ex-situ measurements (i.e., in laboratory air) were performed using either a 20 × or 100 × objective. Spectra were obtained with incident laser powers in the range of ~ 1–10 mW and rejection filters (OptiGrate) were used to reject unshifted laser light. Additionally, a confocal pinhole was used to further reject extraneous light and increase spatial resolution. Spectra reported here were obtained with the 300 lines/mm grating of a Princeton Instruments Acton SP2300 spectrometer coupled with a Pixis 400 detector. The system was calibrated using a neon spectral calibration lamp.

After the in-situ and ex-situ vibrational spectroscopies were completed, backscattered electron images of the collected particulates were obtained using scanning electron microscopy (SEM; FEI, Inspect F model). In backscattered images, particles with higher average atomic numbers appear brighter, meaning particulates containing uranium are easily distinguishable from the KBr surface. Samples were coated with a ~ 10 nm layer of carbon for conductivity. Energy-dispersive X-ray spectroscopy (EDS) was also performed to determine semi-quantitative O/U ratios of the collected particulates.

## Results

### In-situ infrared absorption spectroscopy

**Figure 2:**
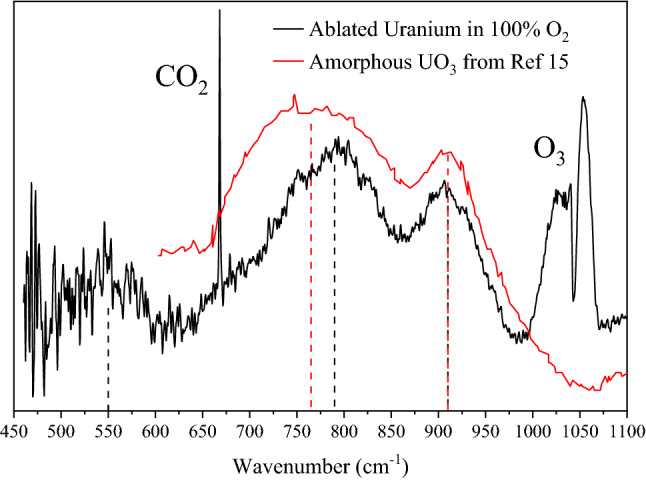
In-situ FTIR spectra of collected particulates after ablation of uranium in 100% O_2_ (black). Shown are three features attributed to am-UO_x_ (where 3 ≤ x ≤ 4) as well as ozone and carbon dioxide (produced during ablation). Also plotted is a spectrum of amorphous UO_3_ digitized from Hoekstra and Siegel (red)^15^. The experimental spectra, consisting of 30 averaged scans at 1 cm^−1^ resolution, were collected ~ 1 min after ablation.

Infrared spectroscopy was the first spectroscopic analysis of particulates formed via LA because IR spectra can distinguish between different molecular phases and the IR beam is sufficiently broad so that a relatively large surface area can be measured in one scan. The FTIR spectrum of the collected particulates after ablation in 100% O_2_ is presented in Fig. 2 from 450 to 1100 cm^−1^. Although KBr allows IR transmission down to ~ 400 cm^−1^, the decreasing sensitivity of the broadband detector at lower wavenumbers makes the signal to noise ratio too low to be useful below ~ 460 cm^−1^. Three features located at 550, 790, and 910 cm^−1^ are observed (indicated by dashed black lines) which are consistent with uranium oxides and are best attributed to amorphous UO_x_ (am-UO_x_) where 3 ≤ x ≤ 4 (see "Discussion" for details). These features are not observed in control experiments in which other metals are ablated in similar atmospheres, consistent with their assignment to uranium containing materials.

For reference, a more complete spectrum from 450 to 3800 cm^−1^ is given in Fig. S1 (Supporting Information); only weak water vapor features are observed above 3800 cm^−1^. Of note, gas-phase bands of ozone (O_3_), CO, and CO_2_ are present in the spectrum (located at 668, 1043, 2112, 2143, and 2349 cm^−1^)^34^. These molecules are synthesized in situ during, or subsequent to, the ablation process. Also observed are features attributed to adsorbed CO_2_^35–38^ at 1262, 1371, and 1536 cm^−1^ as well as adsorbed water^38,39^ with a single broad feature from ~ 3020 to 3670 cm^−1^. The water feature is attributed to adsorption of atmospheric water from laboratory air onto the external surface of the KBr windows that seal the ablation chamber.

**Figure 3:**
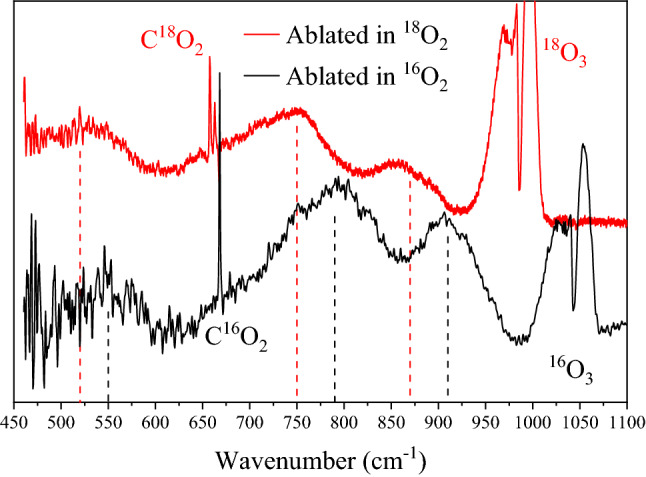
In-situ FTIR spectra of collected particulates after ablation of uranium in 100% O_2_ (black) and ^18^O_2_ (red). The three am-UO_x_ (3 ≤ x ≤ 4) features, indicated with dashed lines, shift to lower frequencies upon ^18^O-substitution, as expected with oxygen-containing molecules. Furthermore, spectral features of ozone and carbon dioxide also shift confirming their formation during ablation. Spectra are offset for clarity.

In Fig. 3, the infrared spectra of particulates collected after ablation in 100% O_2_ are compared with particulates produced in 100% ^18^O_2_. The three features attributed to am-UO_x_, indicated with dashed lines, shift to lower wavenumbers by ~ 30–40 cm^−1^ upon isotopic substitution, specifically to 520, 750, and 870 cm^−1^. As a given vibrational frequency decreases with increasing reduced mass, the observed frequency shifts are evidence that these features are due to oxygen-containing molecules. As seen, the bands for CO_2_ and O_3_ also shift (by ~ 11 and 58 cm^−1^ respectively) which confirms those species are produced by ablation.

**Figure 4:**
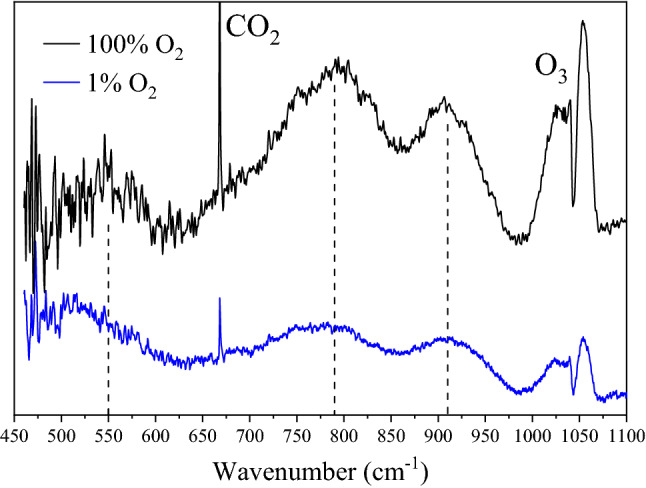
In-situ FTIR spectra of collected particulates after ablation of uranium in 100% O_2_ (black) and 1% O_2_ (blue). Formation of am-UO_x_ (3 ≤ x ≤ 4), indicated by the two uranyl stretching modes (790 and 910 cm^−1^), is observed in both high and low oxygen environments. Additional spectral intensity below 550 cm^−1^ suggests a different uranium oxide, assigned to UO_2_, is also formed in 1% O_2_ atmospheres. Spectra are offset for clarity.

A FTIR spectrum of particulates formed in 1% O_2_/Ar is shown in Fig. 4 along with that of particulates formed in 100% O_2_ for comparison. The presence of the features above 700 cm^−1^ imply am-UO_x_ is still formed in a low oxygen environment; however, the increased signal between 460 and 535 cm^−1^ suggests the formation of a new feature that is convolved with the am-UO_x_ feature at 550 cm^−1^. This new feature resembles that of UO_2_ (see Fig. S2) but is not definitive^40^. This feature, tentatively at 515 cm^−1^, also shifts upon ^18^O substitution by ~ 20 cm^−1^ (Fig. S3), although this value may be larger as the feature is on the edge of the resolved spectrum. The two am-UO_x_ features also shift by ~ 35–40 cm^−1^ upon ^18^O substitution (a similar shift to that observed in 100% O_2_/^18^O_2_). As expected, the intensity of O_3_ and CO_2_ has decreased, most likely due to the limited availability of oxygen during ablation.

### In-situ and ex-situ Raman spectroscopy

**Figure 5:**
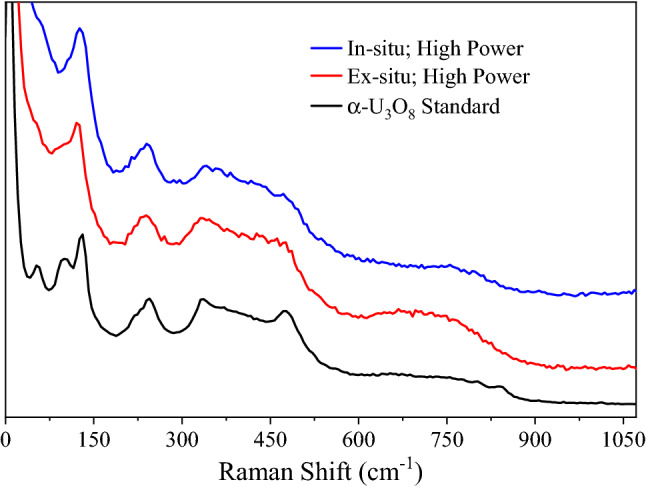
Raman spectra of collected particulates measured in situ (blue) and ex situ (red) using a high HeNe power. Also shown is the spectrum measured from an α-U_3_O_8_ standard (black). Spectral similarities at ~ 125, 240, and 295–520 cm^−1^ suggest that a higher power HeNe beam can convert am-UO_x_ particulates to α-U_3_O_8_. Spectra are offset for clarity.

**Figure 6:**
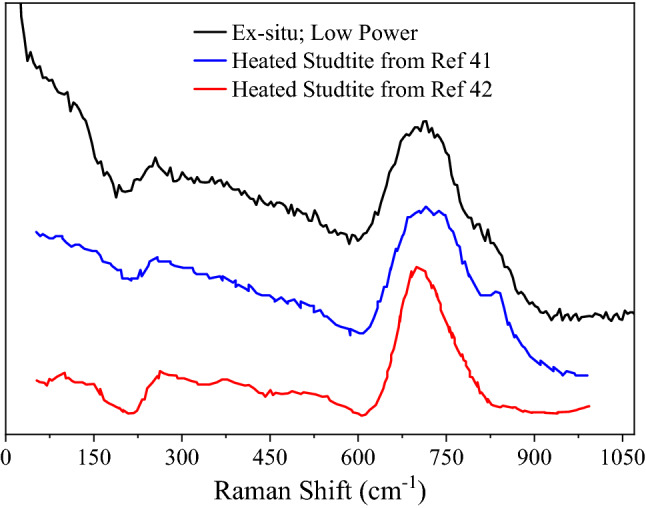
Ex-situ Raman spectra of collected particulates using a low HeNe power (black) plotted along with am-UO_x_ spectra from Thompson et al.^41^ (blue) and Spano et al.^42^ (red). Comparisons give support for the am-UO_x_ assignment of particulates. Spectra are offset for clarity.

As opposed to the relatively large area probed by the IR beam, Raman spectra were collected of individual particulates. Figures 5 and 6 show in-situ and/or ex-situ Raman spectra of the collected particulates formed in 100% O_2_. Spectra were obtained using two different incident powers, hereinafter referred to as “high” and “low”, which correspond to a few milliwatts (“high”) and less than a milliwatt (“low”). Most particulates were observed to be a few micrometers in size and localized near the ablation site. As discussed above, directly after the in-situ infrared measurement the chamber was evacuated and transferred to the Raman system. Spectra of particulates were measured in the following sequence: (1) in situ low power (2) in situ high power (3) ex situ low power and (4) ex situ high power.

Under low power, the particulates did not yield in-situ Raman spectra. Attempting to obtain a spectrum by increasing the HeNe power caused the particulates to break apart changing from circularly shaped to irregular (see Fig. S4). This result was reproduced with every particulate investigated throughout all experiments. It was concluded that the laser deposited enough energy to drive chemistry in the sample particulates. Spectra produced under this higher power (Fig. 5, blue trace) closely resemble α-U_3_O_8_ when compared to a standard (Fig. 5, black trace; Costech Analytical Technologies 080016 CRM 129-A).

Raman spectra of the particulates were also measured ex situ. With a low power HeNe beam (Fig. 6, black trace), the obtained spectrum shows a strong feature at 711 cm^−1^ with a shoulder near 825 cm^−1^. Increased signal intensity from ~ 250 to 600 cm^−1^ (presumably from several weak modes) was also observed which gives the appearance of a dip in the spectrum at ~ 200 cm^−1^. These spectral elements are consistent with am-UO_x_ from literature (Fig. 6, blue and red traces)^41,42^. As with the in-situ results, a high power HeNe produces ex-situ spectra similar to α-U_3_O_8_ (Fig. 5, red trace).

**Figure 7:**
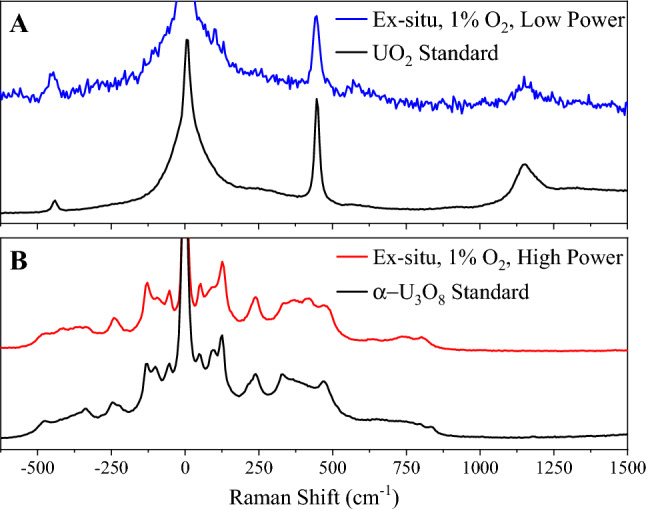
Ex-situ Raman spectra of particulates formed in 1% O_2_/Ar analyzed with a low power (top, A) and high power (bottom, B) HeNe beam. Also shown are spectra for α-U_3_O_8_ and UO_2_ standards. A low power HeNe beam indicates UO_2_ is formed during the ablation process; however, with sufficient heat, particulates can be converted to α-U_3_O_8_. Spectra are offset for clarity.

Figure 7 presents ex-situ Raman spectra of particulates formed during ablation in 1% O_2_/99% Ar. When probed with a low power HeNe beam (Fig. 7A), the spectra resemble that of UO_2_. Spectral features at ~ 445 and 1155 cm^−1^ match those given in literature^13,43^ and a UO_2_ standard plotted as reference (New Brunswick Laboratory, CRM 125-A). A weaker feature around 570 cm^−1^ is also discernible. In order of increasing frequency these bands are assumed to correspond to the fluorite fundamental Raman active vibration, a nominally Raman inactive mode, and an overtone of the latter^44,45^. In Fig. 7B, the spectrum of the same particulate after irradiation with a high power HeNe beam is shown where the conversion to what appears to be α-U_3_O_8_ is again observed. Interestingly, Raman spectra of am-UO_x_ were never observed in the sample collected in 1% O_2_, despite am-UO_x_ being detected in the IR spectrum. This suggests the am-UO_x_ material formed in low oxygen is very fine (sub-μm).

### SEM imaging and EDS analysis

**Figure 8:**
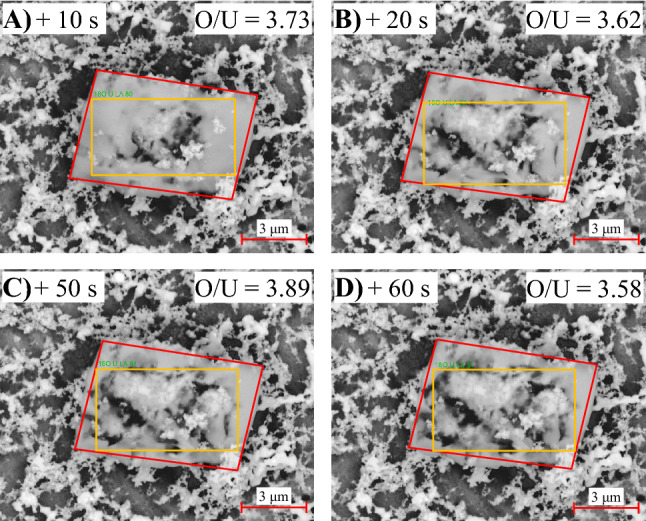
Backscattered SEM/EDS images and O/U ratios of a ~ 8 × 5 μm am-UO_x_ particulate (or collection thereof), where the particulate is outlined in red and the area measured for EDS is outlined in yellow. (**A**)–(**D**) represent images of the particulate after consecutive EDS spectra were measured with collection times of 10, 10, 30, and 10 s, respectively. As seen, the electron beam affected the sample. The measured O/U ratios were between 3.58 and 3.89, consistent with the am-UO_x_ (3 ≤ x ≤ 4) assignment.

Sample images are shown in Fig. 8 where A–D represent increasing increments of dwell time of the electron beam on a particulate formed in 100% O_2_. The red outline gives approximate boundaries of the particles whereas the yellow outline shows the area of EDS measurement. Note, EDS measurements were performed after the vibrational spectroscopies; therefore, the particulates were exposed to laboratory air. When not being analyzed, and not otherwise in the chamber, all KBr collection substrates were kept in a desiccator to discourage reactions with atmospheric water.

## Discussion

Several uranium oxides were considered when determining which phase is produced from the ablation of uranium in an oxygen-rich environment. As seen in Fig. S5, the infrared spectrum measured here does not perfectly match any of the five main crystalline UO_3_ phases (α, β, γ, δ, and ε)^15,46^. Strong similarities exist for α-UO_3_ and β-UO_3_, however, these phases were removed from consideration due to the lack of spectral intensity from 600 to 700 cm^−1^ and above ~ 950 cm^−1^, respectively, in our data. Comparisons of our infrared data to literature spectra of UO_2.9_, U_3_O_8_, U_3_O_7_, and U_4_O_9_ do not provide convincing similarities^14,15,40,46^.

Along with the experimental data obtained here, Fig. 2 includes a spectrum from Hoekstra and Siegel (red trace), which is attributed to amorphous UO_3_^15^. This spectrum shows symmetric and asymmetric^16,41,47,48^ uranyl stretching features at 765 and 910 cm^−1^, respectively. This is in good agreement with the in-situ frequencies measured in this study at 790 and 910 cm^−1^ (Fig. 2, black trace). Previous works studying the thermal decomposition of studtite (UO_4_∙(H_2_O)_4_) to am-UO_x_ (where 3 ≤ x ≤ 4) also report IR features near 750 and 900 cm^−1^^16,39,41^. Thus, there is strong evidence that the three features seen here may be attributed to the symmetric and asymmetric uranyl stretching modes (790 and 910 cm^−1^) and a U–O stretching mode (550 cm^−1^) of am-UO_x_ (where 3 ≤ x ≤ 4). Although, to our knowledge, no spectra of am-UO_x_ have been reported below 600 cm^−1^, many uranium-containing molecules produce U–O stretching modes near 500 cm^−1^ giving additional support for this assignment^46,49^. Furthermore, plume dynamic modeling of laser ablated uranium suggests higher oxides (e.g., UO_3_) will form at the outer edge of the plume-atmosphere interface^50^, in agreement with the observed major products here. As previously mentioned, Koroglu et al.^23^ conclude α-UO_3_ is produced when uranium nitrate and O_2_ are passed through an ICP and allowed to cool. In that study, the millisecond timescale of reactions may allow for UO_3_ to arrange in a crystalline fashion whereas the microsecond timescale of a laser ablation process may only allow amorphous material to form.

As mentioned, ozone is produced from the laser ablation process and it is unclear how ozone affects plasma condensation chemistry, given its oxidative properties. This impact should be considered for similar laser ablation experiments and incorporated into models of such processes. Furthermore, it is expected that metal clusters along with vaporized metal are produced during the ablation because of the nanosecond pulse width; therefore, experiments viewing the effect of laser pulse width on resulting speciation should be performed.

**Figure 9:**
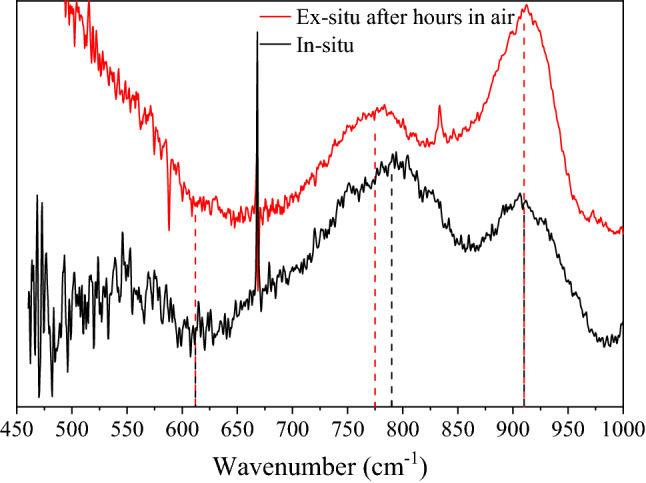
FTIR spectra of collected particulates after ablation of uranium in 100% O_2_ measured in situ (black) and ex situ after hours in air (red). The uranyl symmetric stretching mode located at 790 cm^−1^ shifts to 775 cm^−1^ with the other modes appearing to be unaffected. The new location of this feature is consistent with that commonly reported in literature. Spectra are offset for clarity.

The approximately 25–40 cm^−1^ difference in the symmetric stretching mode frequency between our work and literature (Fig. 2) might be explained by the absence or presence of adsorbed water. FTIR spectra from literature are often collected in air and show residual adsorbed water whereas the am-UO_x_ formed here is nominally completely anhydrous as it is formed from atoms under vacuum. To support this, FTIR spectra were taken of the samples after being exposed to air for several hours which show a change in the symmetric stretching mode from 790 to 775 cm^−1^; the two other modes appear unchanged (Fig. 9). Furthermore, the observed increase in intensity of the asymmetric stretching mode is indicative of am-UO_x_ hydration^16,39^. This result suggests the true frequency for the symmetric stretching mode of solid-state am-UO_x_ is slightly higher than that commonly reported in literature.

Unfortunately, in-situ Raman spectra could not be obtained with low power to support the am-UO_x_ assignment; however, the ex-situ Raman spectra obtained with a low HeNe power (Fig. 6) matches very well with reported spectra of am-UO_x_ (obtained after heating studtite)^41,42^. As hydration was observed in the FTIR spectra, it is important to note that the ex-situ Raman spectra are of this hydrated material. With a high HeNe power, the am-UO_x_ particles always converted to α-U_3_O_8_, a thermal process well documented in literature^16,38,39,41^. This transition has an intermediate step where α-UO_3_ is formed, however, this stage was never detected. It is assumed the small particle sizes cause poor heat dissipation which is why the conversion to α-U_3_O_8_ is observed. Raman spectra of U_4_O_9_ is similar to α-U_3_O_8_; however, observed spectral intensity at 239 cm^−1^ and between 300 and 400 cm^−1^ (Fig. 5) are indicative of α-U_3_O_8_^13,51,52^, and our measured spectra compares closely to an α-U_3_O_8_ standard. Although there is an observed temperature dependence on peak relative intensities and broadness for the α-U_3_O_8_ standard (Fig. S6), it is still clear that the material formed from ablation is a convincing match. Of note, Raman spectra of UO_2_ or crystalline UO_3_ were never observed from particulates formed in 100% O_2_. Also, α-U_3_O_8_ spectra were only ever measured with the high HeNe power.

SEM images of potential am-UO_x_ particulates were inconclusive as the electron beam altered the particulates of interest. These particulates appeared bright relative to the KBr substrate; however, as seen in Fig. 8A, after focusing onto the particle and collecting a 10 s EDS spectrum, the original particulate had a relatively darker center. This effect became more distinct after one minute of beam interaction with the particulate (images B-D). The average O/U ratio of the four spectra obtained by EDS is ~ 3.7 ± 0.1. Although the uncertainties are expected to be relatively large, this result further supports the assignment of UO_x_ (3 ≤ x ≤ 4). It is difficult to determine what is happening to the particulate under the electron beam. At a minimum, some of the material moved during the analysis as the relatively dark KBr substrate became more apparent with increasing dwell time of the electron beam on the particulate. This could explain why the O/U ratio was not largely affected but a change was observed. The results, however, do not exclude the possibility of polymorphic changes.

Ablation of uranium in a low oxygen environment (1% O_2_/Ar) produces both am-UO_x_ and UO_2_. Due to the limitation of KBr and the MCT detector, the presence of UO_2_ cannot be confirmed via infrared data alone as its main spectral feature is from 250 to 600 cm^−1^^40,46^; however, Raman spectra indicate UO_2_ is clearly formed (Fig. 7A). This was also observed by Koroglu et al.^23^ where the predominant oxide formed in a relatively low O_2_ atmosphere is UO_2_ not UO_3_. The Raman spectrum of UO_2_ presented in Fig. 7 shows that the antistokes/stokes intensity ratio of the fluorite band is larger for particles formed from laser ablation when compared to a UO_2_ standard. This effect is consistent with heating of the sample. Although not the focus of this study, the minimum temperature for UO_2_ → α-U_3_O_8_ conversion could be obtained from such measurements (after carefully correcting for the wavelength sensitivity of the instrument over the corresponding spectral range).

The Raman spectrum of particulates formed from the ablation of uranium in 1% ^18^O_2_/Ar is shown in Fig. S7 along with that of UO_2_ formed from ablation in 1% ^16^O_2_/Ar for comparison. Although the features are weak, a shift of ~ 14 cm^−1^ is observed. This frequency change is less than that reported by Lv et al.^53^ who show an isotopic shift of 22 cm^−1^ (from 441 to 419 cm^−1^). Scaling the frequency from the change in reduced mass using υ ∝ sqrt(1/μ), the approximate shift is 24 cm^−1^. This suggests the features observed here might be caused by an isotopically mixed ^16^O/^18^O system, which could stem from the exchange with laboratory air.

## Conclusion

To determine the influence of oxygen on rapidly cooling uranium, laboratory-scale experiments were performed investigating the laser ablation of depleted uranium in 100% O_2_ and 1% O_2_/Ar. Particulates formed during this process were analyzed using infrared absorption and Raman spectroscopies, measured in-situ and ex-situ, which yields uranium speciation information relevant to nuclear forensics. Data show am-UO_x_ (3 ≤ x ≤ 4) is produced from the ablation of uranium in 100% O_2_. Comparisons to literature spectra of am-UO_x_ (formed from heating studtite) imply the symmetric stretching frequency is slightly higher than what is commonly reported; we interpret this as an effect of hydration. Ablation in a low, 1% O_2_/Ar environment results in formation of UO_2_ as the primary phase, although am-UO_x_ is still observed. Furthermore, am-UO_x_ and UO_2_ convert to α-U_3_O_8_ when sufficient heat is added. This work improves our understanding of gas phase chemical reactions between uranium and oxygen, which can inform models of nuclear explosions to improve our predicative capabilities of particle formation and transport. Using these results as a basis, future work could investigate the speciation of vaporized uranium alloys or uranium compounds (e.g., oxides, carbides, and nitrides). Such data would be useful for modeling incidents at nuclear power plants where those materials are often used. Finally, we note that more energetic, longer pulse duration lasers could be used to probe additional cooling timescales. This larger dataset, combined with literature viewing millisecond cooling, can be used to extrapolate to cooling regimes relevant to nuclear fireball chemistry.

## Supplementary Information

Supplementary Information.
