# Supplementary material for: The effect of oxygen concentration on the speciation of laser ablated uranium
Source: Sci Rep. 2022 Mar 7;12:4030. doi: 10.1038/s41598-022-07834-9 (PMC8901731; doi:10.1038/s41598-022-07834-9)
Supplement: Supplementary file 1 — Supplementary Information. [file 41598_2022_7834_MOESM1_ESM.pdf]

The Effect of Oxygen Concentration on the Speciation of Laser Ablated Uranium

Mark A. Burton*[,a], Alex W. Auner[a], Jonathan C. Crowhurst[a], Peter S. Boone[a], Lauren A. Finney[b], David G. Weisz[a], Batikan Koroglu[a], Igor Jovanovic[b], Harry B. Radousky[a], Kim B. Knight[a]

*Corresponding author email: burton34@llnl.gov

[a] Lawrence Livermore National Laboratory
7000 East Ave
Livermore, CA 94550, USA

[b] Department of Nuclear Engineering & Radiological Sciences, University of Michigan
2355 Bonisteel Blvd
Ann Arbor, MI 48109, USA

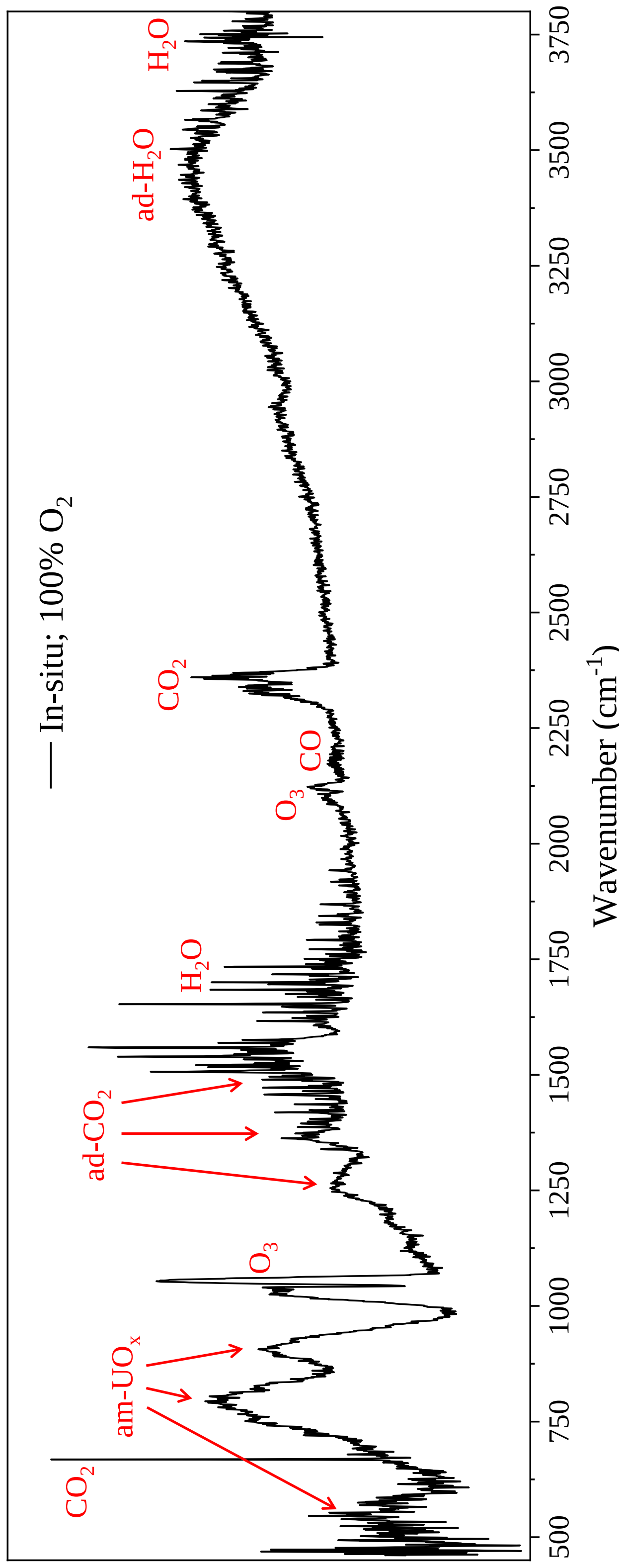

Figure S1: In-situ FTIR spectrum taken of particulates formed via laser ablation of uranium in 100% O_2 consisting of 30 averaged scans at 1 cm^{-1} resolution from 450 – 3800 cm^{-1}. Gas-phase bands of O_3, CO, and CO_2 are present in the spectrum (synthesized during ablation) as well as features attributed to amorphous $UO_{3\text{-}4}$, adsorbed CO_2, and adsorbed water.

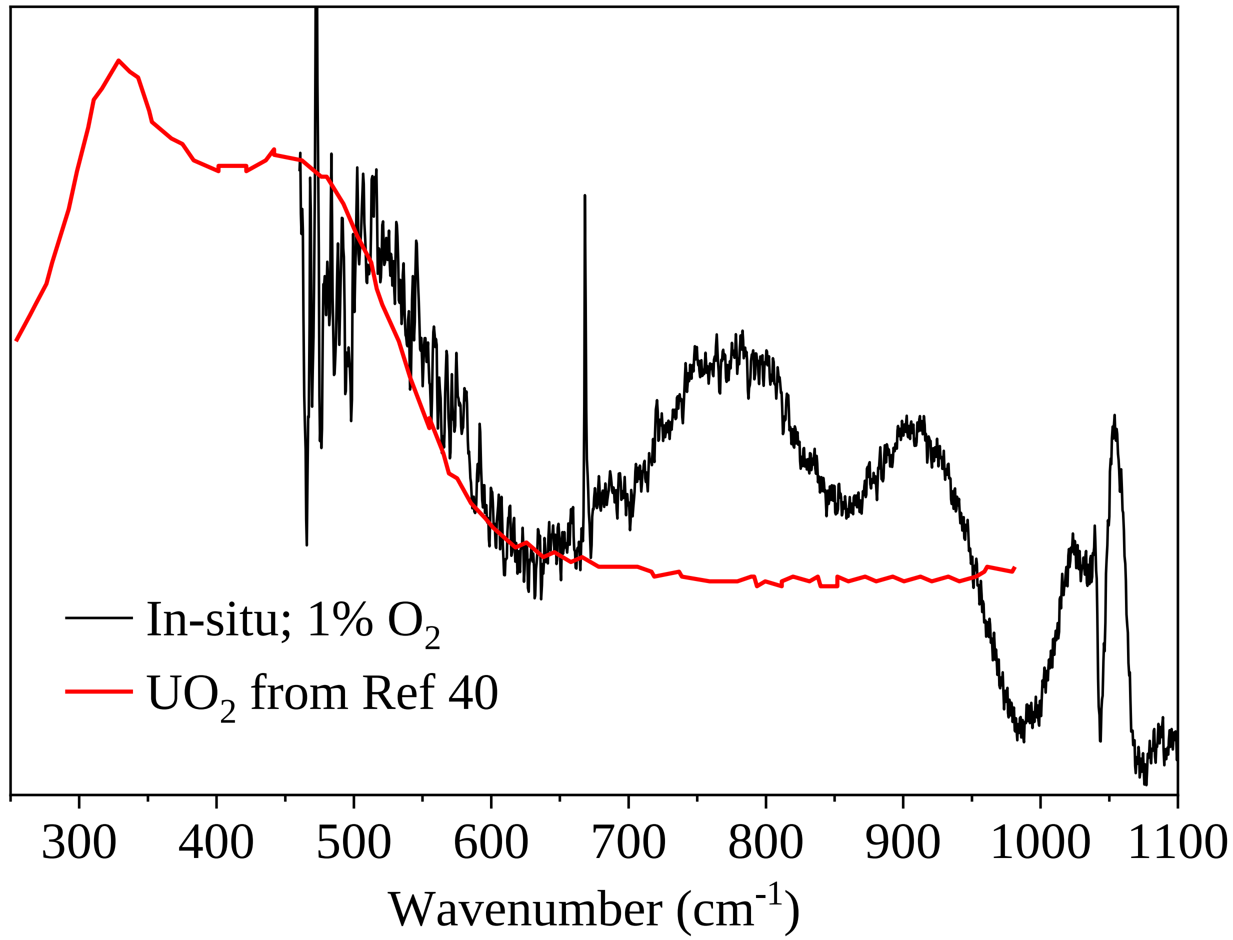

Figure S2: In-situ FTIR spectra of collected particulates after ablation of uranium in 1% O_2 (black); also plotted is a spectrum of UO_2 digitized from Allen et al.[40] (red). Comparisons with literature as well as corresponding Raman spectra yield the assignment of UO_2 as the low frequency feature. The two features above 700 cm^{-1} are am-UO_x ($3 \leq x \leq 4$).

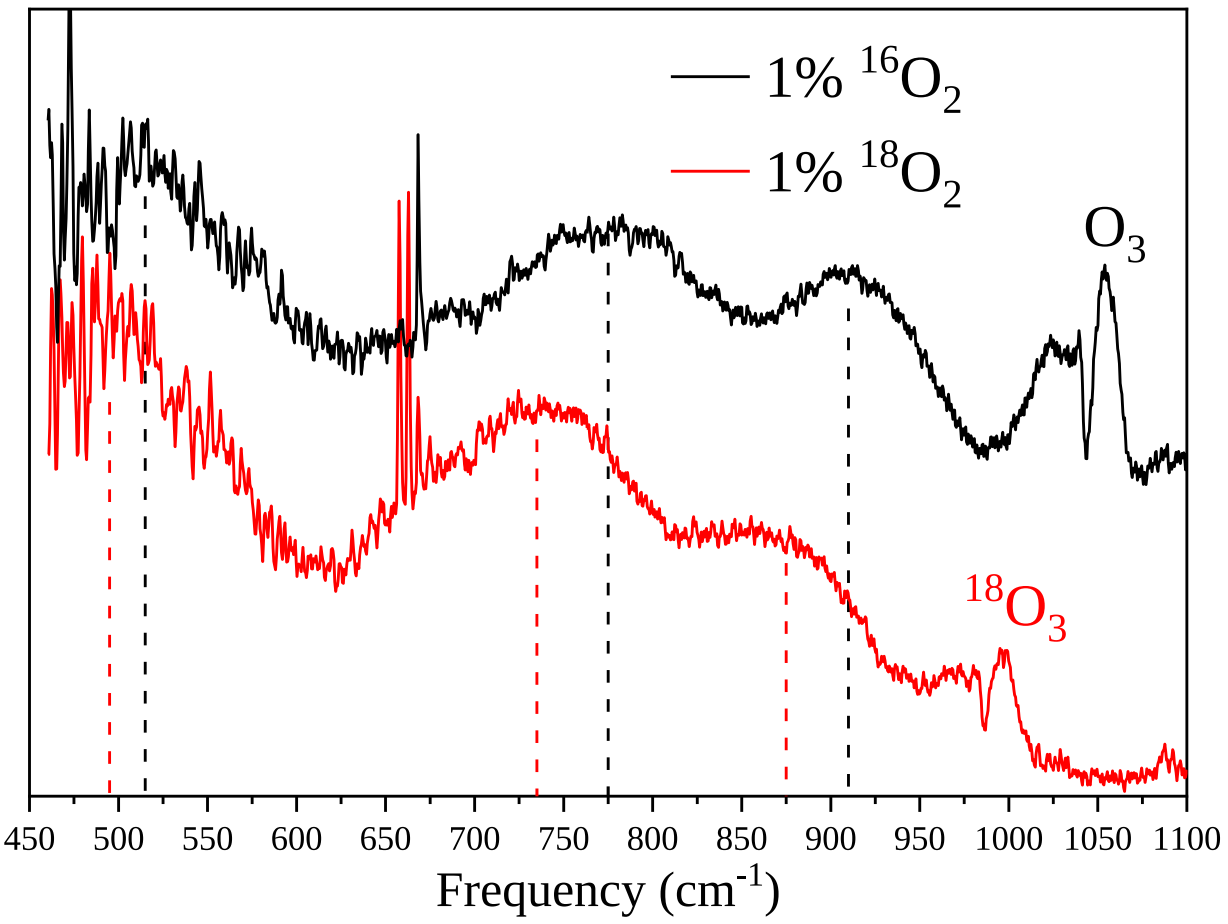

Figure S3: In-situ FTIR spectra of collected particulates after ablation of uranium in 1% O_2 (black) and 1% $^{18}O_2$ (red). The two am-UO_x features above 700 cm^{-1} shift by ~35 – 40 cm^{-1} (consistent with that observed from data collected in 100% O_2). The UO_2 feature shifts by ~20 cm^{-1}; however, this valve could be larger. Spectra are offset for clarity.

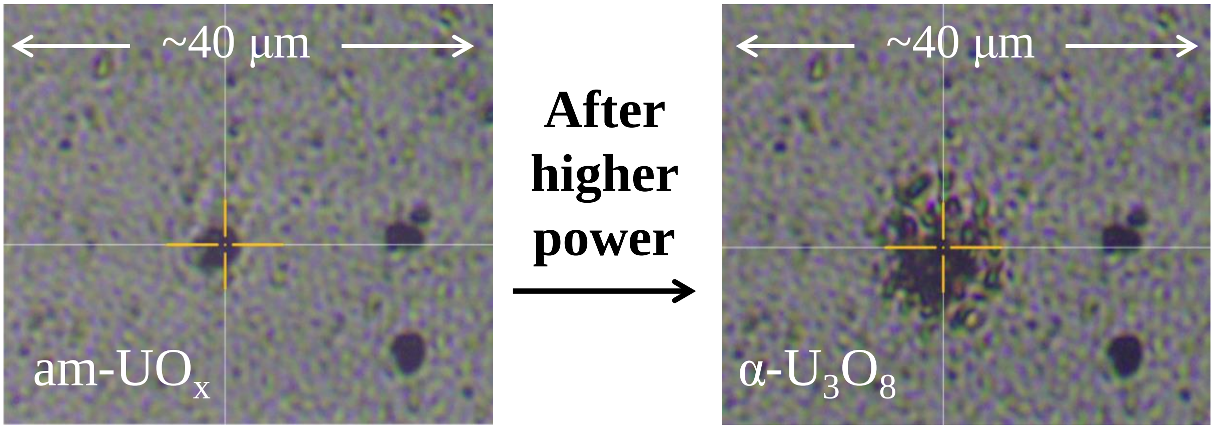

Figure S4: Optical images of KBr surface showing backlit particles formed from the ablation of depleted uranium in 100% O_2. Particulates, initially small and circular am-UO_x, transform instantaneously into irregular and distributed α-U_3O_8 after irradiation with a relatively high HeNe power.

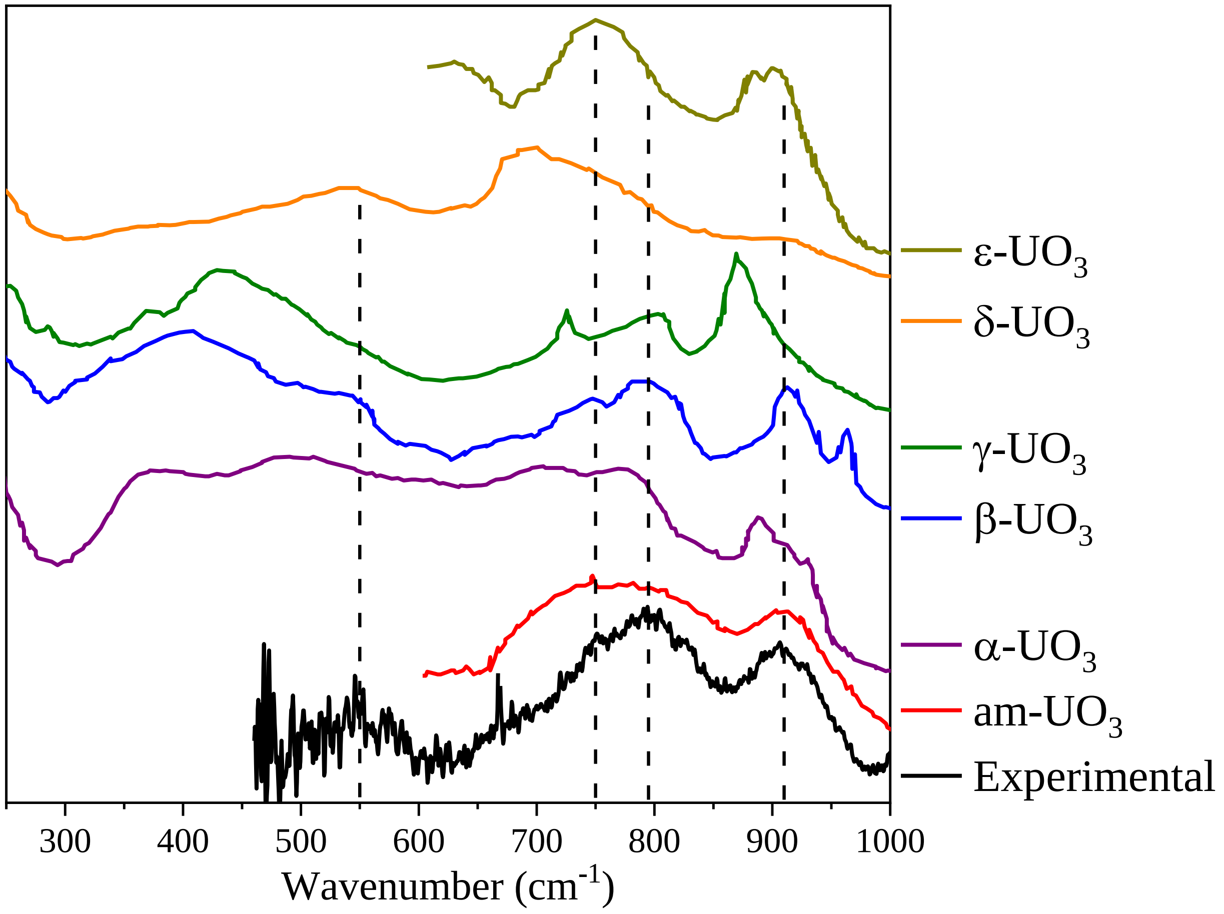

Figure S5: Comparison between the experimental infrared spectrum of particulates collected from the ablation of depleted uranium in 100% O_2 to spectra of the five crystalline UO_3 phases (α, β, γ, δ, and ε) taken from Allen and Homes (1994)[46] as well as Hoekstra and Siegel (1961)[15]. There are similarities to α-UO_3 and β-UO_3, however, spectra for these phases differ from the observed spectra collected here in the 600 – 700 cm^{-1} and above ~950 cm^{-1} regions. Spectra are offset; the sharp CO_2 peak at ~668 cm^{-1} was removed from the experimental data for clarity.

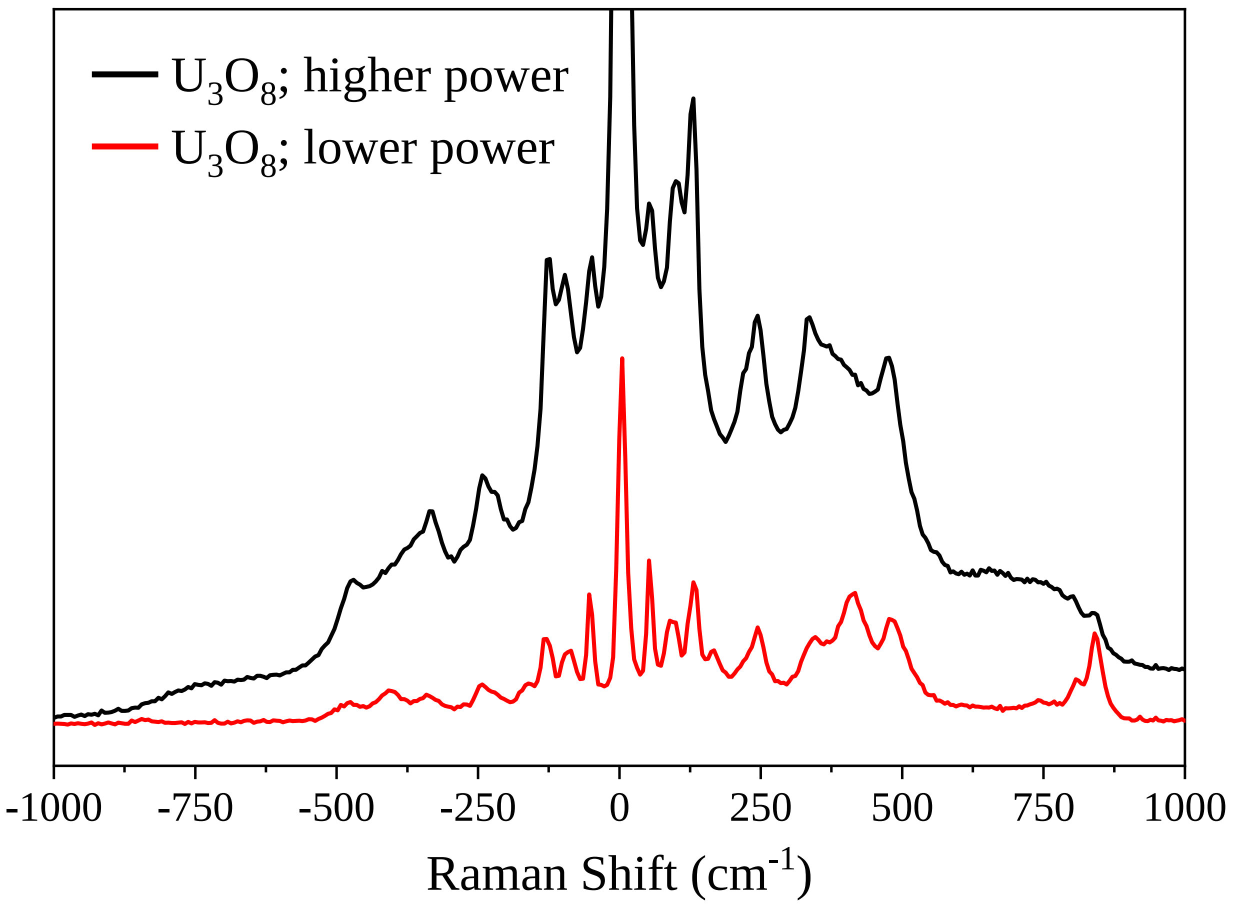

Figure S6: Raman spectra of an α-U_3O_8 standard taken with different incident laser powers. The relatively lower power beam produces spectral features that are sharper and more defined when compared to spectra taken with a higher power beam. This is most likely a temperature effect.

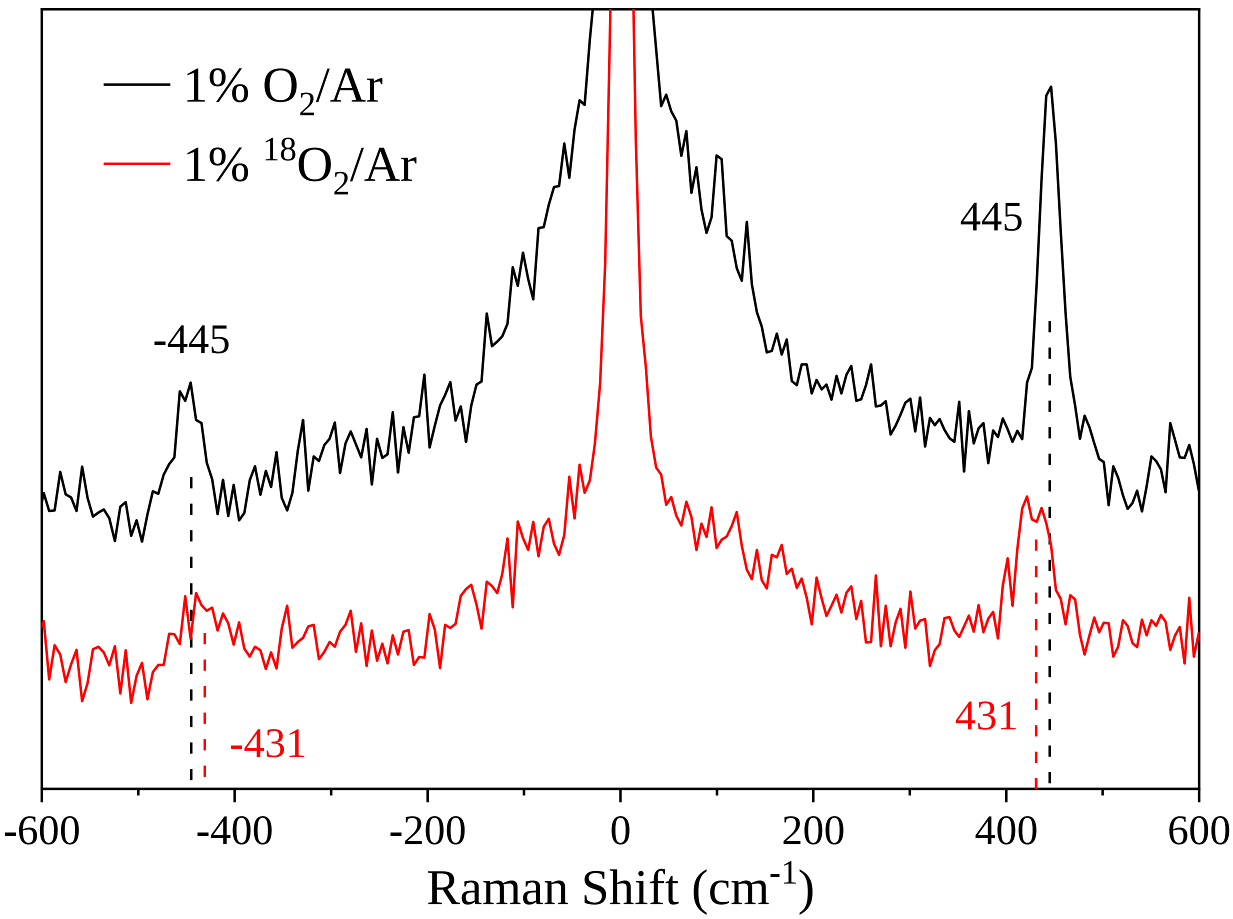

Figure S7: Ex-situ Raman spectra of particulates formed in 1% $^{18}O_2$/Ar analyzed with a low power HeNe beam. Also shown is a spectrum of particulates formed in 1% O_2/Ar (Figure 7 top) for reference. An isotopic shift of ~14 cm^{-1} is observed upon ^{18}O substitution. Comparison with literature spectra suggest these particulates may contain both ^{16}O and ^{18}O atoms. Spectra are offset for clarity.
